# Association between aortic stiffness, carotid vessel wall thickness and stenosis severity in peripheral arterial occlusive disease: a comprehensive MRI study

**DOI:** 10.1186/1532-429X-14-S1-P132

**Published:** 2012-02-01

**Authors:** Harrie van den Bosch, Jos J Westenberg, Lucien E Duijm, Alette Daniels-Gooszen, Gwat Yoe M The, Joep A Teijink, Albert de Roos

**Affiliations:** 1 Radiology, Leiden University Medical Center, Leiden, Netherlands; 2 Radiology, Catharina Hospital, Eindhoven, Netherlands; 3 Vascular Surgery, Catharina Hospital, Eindhoven, Netherlands

## Summary

Aortic pulse wave velocity sampled in the descending aorta is associated with maximal stenosis severity, visually scored on CE-MRA in patients with PAOD whereas stenosis severity is correlated to a lesser extent with carotid vessel wall.

## Background

In atherosclerosis, arterial wall thickening and stiffening precede luminal narrowing. MRI is well-validated for imaging vessel wall thickness (VWT) and stiffness expressed in pulse wave velocity (PWV, defined as the propagation speed of the pressure or flow wave through the aorta). Contrast-enhanced MR angiography (CE-MRA) has evolved into a reliable tool for stenosis detection in peripheral arterial occlusive disease (PAOD). The purpose of this study was to use a comprehensive 3T MRI-approach for comparing stenosis severity on CE-MRA with VWT, sampled in the common carotid artery, and PWV, sampled in the descending aorta.

## Methods

**Figure 1:**
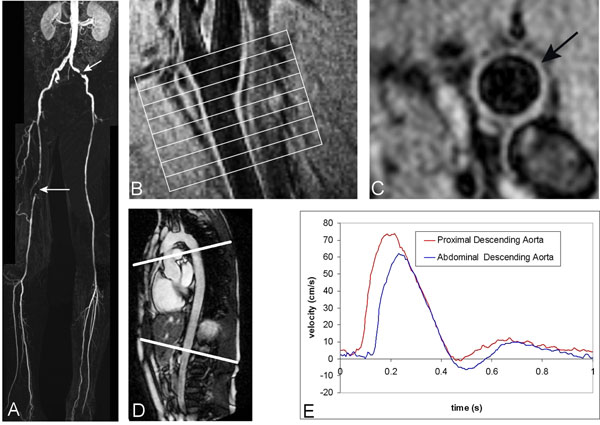
(A) Coronal contrast-enhanced MR angiographic maximum intensity projection images of 67-year-old man presenting with bilateral claudication and significant stenosis in the left external iliac artery (short arrow) and an occlusion in the right superficial femoral artery (long arrow); (B) Sagittal black-blood image of the carotid bifurcation presenting positioning of carotid vessel wall sampling in the common carotid artery (C); (D) Sagittal image of the aorta presenting positioning of two one-directional through-plane velocity-encoded MRI acquisitions at the proximal and abdominal descending aorta. Pulse Wave Velocity is determined from wave propagation analysis between measurement sites (E).

Forty-two patients (23 men; mean age 64±10years) with clinically suspected PAOD were included. Standardized single-injection 3-station moving-table CE-MRA, carotid vessel wall imaging and PWV-assessment were performed at 3T MRI (Philips). With CE-MRA, the arterial tree was evaluated from infrarenal aorta down to the tibial and peroneal arteries (Figure 1A). Visual stenosis classification was performed in consensus by two radiologist in blinded manner using the following categories: class 1 (0%-stenosis), 2 (1-50%), 3 (51-75%), 4 (76-99%) and 5 (100%).

Four transverse images of the common carotid artery were obtained by multi-slice 2D dual inversion recovery black-blood (DIR) fast gradient-echo (Figure 1B) with spectral selective fat suppression. A flexible 2-element surface coil was used and positioned on the neck. Inner and outer lumen contours were manually determined (Figure 1C), defining measurement of mean vessel wall area (VWA) per slice.

PWV was assessed for the descending aorta by applying two one-directional through-plane velocity-encoded MRI acquisitions, planned perpendicular to the aorta and transecting the proximal and abdominal descending aorta, respectively (Figure 1D). PWV was obtained from systolic wave propagation analysis based on the transit-time method (Figure 1E).

PWV was compared with carotid VWA indexed for body surface area and maximal stenosis severity class detected with CE-MRA.

## Results

**Figure 2:**
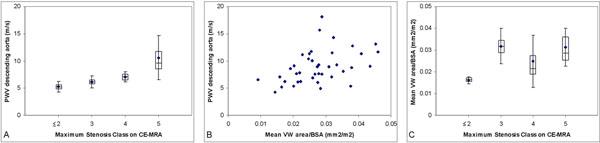
(A) Box-plot and association between maximal stenosis class, visually scored on contrast-enhanced MR angiography (CE-MRA), and pulse wave velocity (PWV) sampled in the descending aorta. Correlation was expressed by Spearman correlation 0.63 (p<0.001); (B) Association between mean vessel wall (VW) area per slice sampled in the common carotid artery, indexed for body surface area (BSA), and PWV. Correlation was expressed by Pearson correlation 0.48 (p=0.002); (C) Box-plot and association between maximal stenosis class and mean vessel wall (VW) area indexed for BSA. Correlation was expressed by Spearman correlation 0.43 (p=0.005).

Mean Fontaine class was 2.3±0.6. Maximal stenosis class per patient presented on CE-MRA was as follows: 2 patients without stenosis (class 1), 1 patient with class 2, 2 patients with class 3, 9 patients with class 4 and 28 patients with class 5. PWV in the descending aorta was well-correlated with maximal stenosis class (Spearman correlation 0.63 (p<0.001), Figure 2A). Carotid VWA and PWV (Pearson correlation 0.48 (p=0.002), Figure 2B) and carotid VWA and maximal stenosis class (Spearman correlation 0.43 (p=0.005), Figure 2C) were correlated to a lesser extent.

## Conclusions

PWV in the descending aorta is associated with maximal stenosis severity, visually scored on CE-MRA in patients with PAOD whereas stenosis severity is correlated to a lesser extent with carotid vessel wall.

## Funding

None.

